# Co-Stimulation with TWEAK and TGF-β1 Induces Steroid-Insensitive TSLP and CCL5 Production in BEAS-2B Human Bronchial Epithelial Cells

**DOI:** 10.3390/ijms252111625

**Published:** 2024-10-29

**Authors:** Sumiko Abe, Norihiro Harada, Yuuki Sandhu, Hitoshi Sasano, Yuki Tanabe, Shoko Ueda, Takayasu Nishimaki, Yoshihiko Sato, Tomohito Takeshige, Sonoko Harada, Hisaya Akiba, Kazuhisa Takahashi

**Affiliations:** 1 Department of Respiratory Medicine, Juntendo University Faculty of Medicine and Graduate School of Medicine, Tokyo 113-8421, Japan; su-abe@juntendo.ac.jp (S.A.); ysando@juntendo.ac.jp (Y.S.); h-sasano@juntendo.ac.jp (H.S.); yutanabe@juntendo.ac.jp (Y.T.); sk-ueda@juntendo.ac.jp (S.U.); t-nishimaki@juntendo.ac.jp (T.N.); yo-sato@juntendo.ac.jp (Y.S.); takeshig@juntendo.ac.jp (T.T.); snharada@juntendo.ac.jp (S.H.); kztakaha@juntendo.ac.jp (K.T.); 2 Research Institute for Diseases of Old Ages, Juntendo University Faculty of Medicine and Graduate School of Medicine, Tokyo 113-8421, Japan; 3 Atopy (Allergy) Research Center, Juntendo University Faculty of Medicine and Graduate School of Medicine, Tokyo 113-8421, Japan; 4 Department of Immunology, Juntendo University Faculty of Medicine and Graduate School of Medicine, Tokyo 113-8421, Japan; hisaya@juntendo.ac.jp

**Keywords:** asthma, EMT, TWEAK, TGF-β1, TSLP, CCL5, MKP-1

## Abstract

Steroid-resistant asthma is a common cause of refractory asthma. Type 2 inflammation is the main inflammatory response in asthma, and the mechanism underlying the steroid-resistance of type 2 inflammation has not been completely elucidated. Tumor-necrosis-factor-like apoptosis-inducing factor (TWEAK) and transforming growth factor (TGF)-β1 are involved in epithelial–mesenchymal transition (EMT) and the production of thymic stromal lymphopoietin (TSLP) and C-C motif chemokine ligand 5 (CCL5). We herein hypothesize that the combined exposure to TWEAK and TGF-β1 may result in the development of steroid resistance in bronchial epithelial cells. The bronchial epithelial cell line BEAS-2B was cultured with or without TGF-β1 or TWEAK, in the presence or absence of dexamethasone (DEX). The roles of Smad-independent pathways and MAP kinase phosphatase 1 (MKP-1) were also explored. Co-stimulation of TWEAK and TGF-β1 induced E-cadherin reduction, N-cadherin upregulation, and TSLP and CCL5 production, which were not suppressed by DEX. Inhibition of the nuclear factor kappa beta (NF-κB) and mitogen-activated protein kinase pathways downregulated steroid-unresponsive TSLP and CCL5 production, whereas knockdown of MKP-1 improved steroid-unresponsive TSLP production, induced by co-stimulation with TWEAK and TGF-β1. Therefore, co-stimulation with TWEAK and TGF-β1 can induce the steroid-insensitive production of TSLP and CCL5 in the bronchial epithelium and may contribute to airway inflammation.

## 1. Introduction

Steroid-resistant asthma is a common cause of refractory asthma. Patients with severe asthma exhibit a limited response to glucocorticoid treatment, requiring the administration of relatively high doses for effective management. This presents considerable challenges in regard to clinical care. Although necessary, the use of high steroid doses may result in heightened side effects and limited clinical benefits. Type 2 inflammation is the primary inflammation type in asthma. However, the mechanisms underlying the development of steroid resistance in type 2 inflammation remains largely unexplored [1,2,3,4]. Non-type-2 inflammation is inherently steroid resistant [5]. As has been previously reported in mouse studies, the coexistence of Th2 inflammation, which is representative of type 2 inflammation, and Th17 cell-mediated inflammation, which represents non-type-2 inflammation and exhibits steroid resistance, induces steroid resistance not only in non-type-2 inflammation but also in type 2 inflammation [6,7]. Our previous report, using cultured bronchial epithelial cells, showed that stimulation with transforming growth factor β1 (TGF-β1)—a cytokine known to induce epithelial–mesenchymal transition (EMT) in bronchial epithelial cells and act downstream of type 2 inflammation—along with the non-type-2 inflammatory cytokine tumor-necrosis-factor-like apoptosis-inducing ligand (TWEAK), enhanced EMT and induced the production of thymic stromal lymphopoietin (TSLP) [8,9]. Moreover, co-stimulation with chitin, which induces non-type-2 inflammation, and ovalbumin, which induces type 2 inflammation, induces steroid-resistant asthma in our mouse model [10]. Based on these facts, we hypothesized that stimuli eliciting non-type-2 inflammation may bestow steroid resistance upon type 2 inflammation in bronchial epithelial cells.

In the present study, we explored the impact of co-stimulation with TWEAK and TGF-β1 on the generation of corticosteroid-unresponsive type-2-inflammation-related cytokines in cultured BEAS-2B bronchial epithelial cells.

## 2. Results

### 2.1. EMT and Cytokine Production Induced by Co-Stimulation with TWEAK and TGF-β1 Were Steroid Unresponsiveness

**Figure 1:**
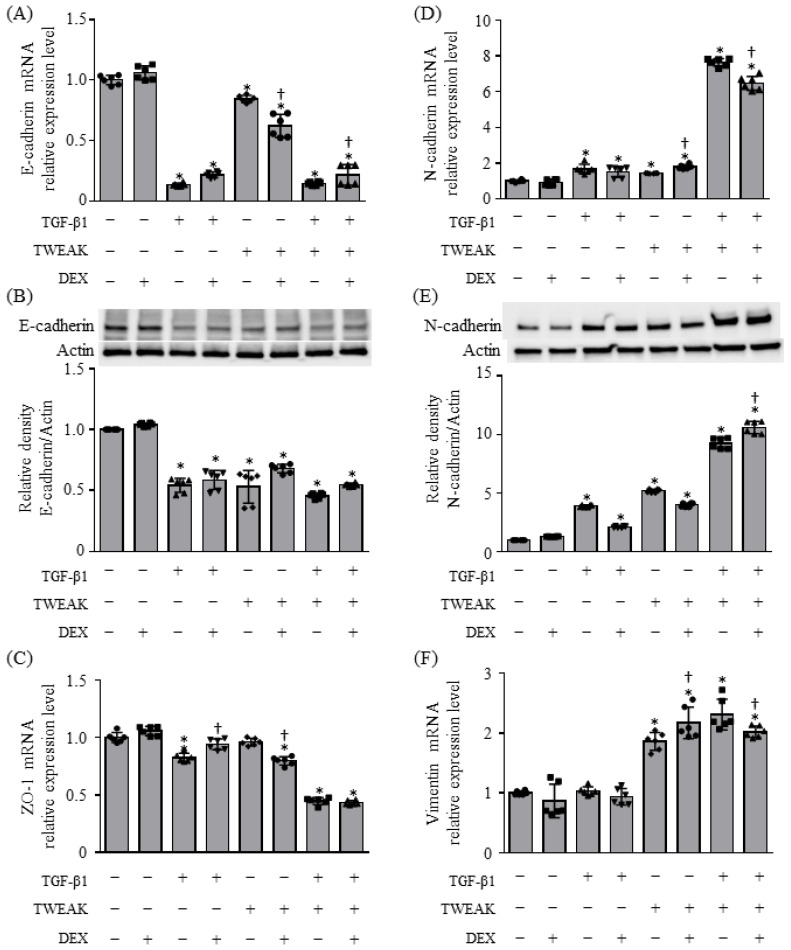
Epithelial–mesenchymal transition (EMT) induced by co-stimulation with tumor-necrosis-factor-like apoptosis-inducing factor (TWEAK) and transforming growth factor (TGF)-β1 result in steroid unresponsiveness. The mRNA levels of *E-cadherin* (**A**), *ZO-1* (**C**), *N-cadherin* (**D**), and *vimentin* (**F**) after 48 h of treatment analyzed by quantitative real-time reverse transcription–polymerase chain reaction (qRT–PCR). Data represent mean ± standard deviation (SD) of two independent experiments. Immunoblotting for E-cadherin (**B**, upper panel) and N-cadherin (**E**, upper panel) expression. Data represent mean ± SD of two independent experiments. * *p* < 0.05, compared to untreated cultures as controls; † *p* < 0.05 compared with cultures in the absence of dexamethasone (DEX).

The TWEAK receptor fibroblast growth-factor-inducible 14 (Fn14) is expressed in BEAS-2B bronchial epithelial cells, and the co-stimulation of TWEAK and TGF-β1 enhances TGF-β1-induced EMT, leading to the production of TSLP, RANTES (regulated upon activation, normal T cell expressed and secreted)/C-C motif chemokine ligand (CCL) 5, and TARC (thymus and activation-regulated chemokine)/CCL17 [8,9]. First, we determined whether co-stimulation with TWEAK and TGF-β1 renders BEAS-2B cells unresponsive to steroids, in terms of EMT and cytokine production. Confluent monolayers of BEAS-2B cells were treated with TWEAK (100 ng/mL) and TGF-β1 (10 ng/mL) with or without DEX (10^⁻⁶^ M). Co-stimulation with TWEAK and TGF-β1 resulted in a reduction in the epithelial marker E-cadherin at both the mRNA and protein levels, a decrease in *ZO-1* expression at the mRNA level, and an increase in the mesenchymal marker N-cadherin at both the mRNA and protein levels, as well as an elevation in vimentin expression at the mRNA level (Figure 1). These regulatory changes were not inhibited by DEX (Figure 1).

**Figure 2:**
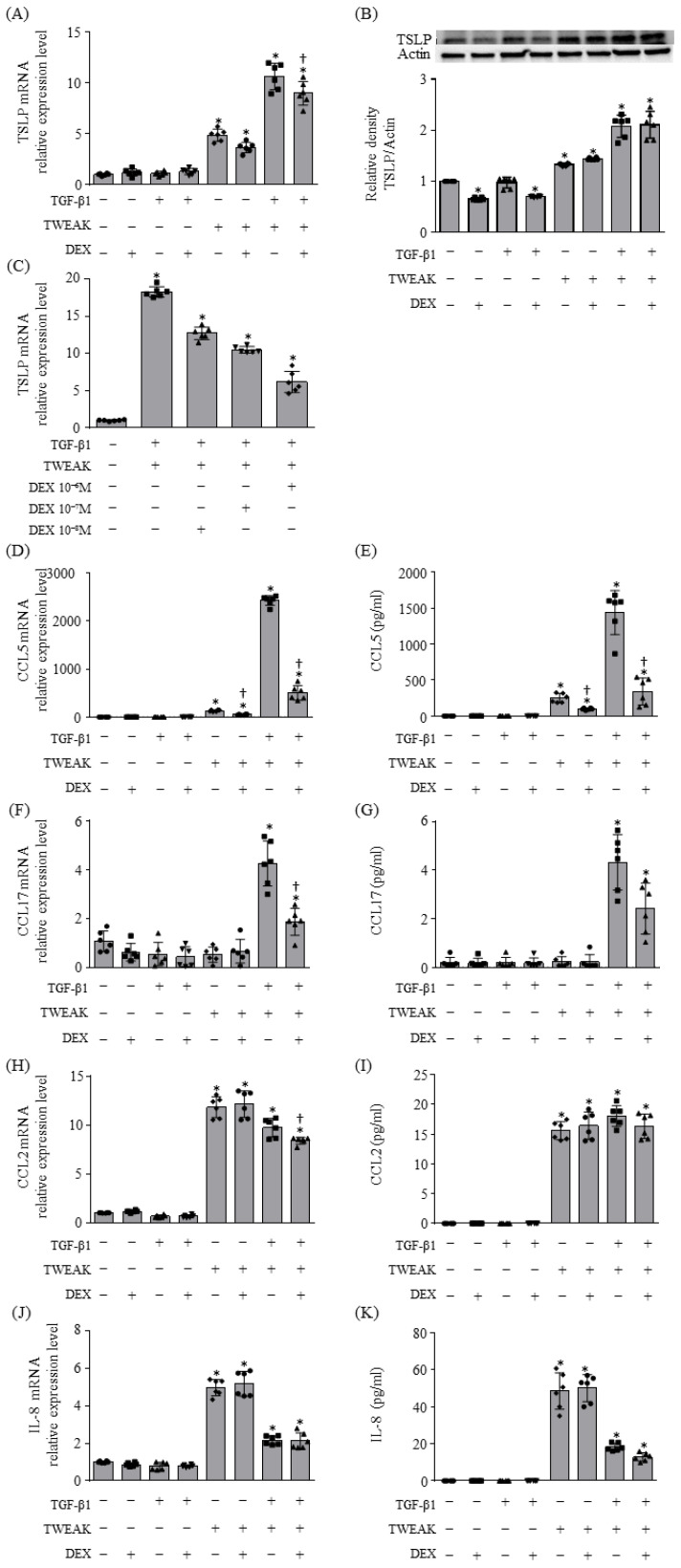
Cytokine production induced by co-stimulation with TWEAK and TGF-β1 were steroid unresponsiveness. The mRNA levels of *TSLP* (**A**,**C**), *CCL5* (**D**), and *CCL17* (**F**) after 48 h of treatment and *CCL2* (**H**) and *IL-8* (**J**) after 2 h of treatment, analyzed by qRT–PCR. Data represent mean ± SD of two independent experiments. TSLP levels after 48 h of treatment assessed by immunoblotting (**B**, upper panel). The density of each band quantified by densitometry using ImageJ (version 6.1) (**B**, lower). The levels of CCL5 (**E**) and CCL17 (**G**) after 48 h of treatment, and CCL2 (**I**) and IL-8 (**K**) after 2 h of treatment in cell culture supernatants, analyzed by enzyme-linked immunosorbent assay (ELISA). Data represent the mean ± SD of three independent experiments. * *p* < 0.05, compared to untreated cultures as controls; † *p* < 0.05 compared with cultures in the absence of DEX.

The production of TSLP and CCL5 in BEAS-2B cells, induced by co-stimulation with TWEAK and TGF-β1, was not entirely suppressed by DEX treatment at either the mRNA or protein levels (Figure 2A,B,D,E). While DEX dose-dependently suppressed *TSLP* mRNA expression during co-stimulation with TWEAK and TGF-β1, the suppression was not complete at a DEX concentration of even 10⁻⁶M, leading us to adopt this concentration for further studies (Figure 2C). Furthermore, both the mRNA and protein levels of CCL17 were upregulated by co-stimulation with TWEAK and TGF-β1 and were not fully suppressed by steroid treatment (Figure 2F,G). Similarly, the mRNA and protein levels of monocyte chemoattractant protein-1 (MCP-1)/CCL2 and interleukin (IL)-8 were upregulated by TWEAK alone or by co-stimulation with TWEAK and TGF-β1, and they were not suppressed by steroid treatment, with the exception of *CCL2* mRNA expression induced by co-stimulation (Figure 2H–K). Additionally, we investigated whether co-stimulation with TWEAK and TGF-β1 could induce steroid-unresponsive mRNA expression of *TSLP* and *CCL5* in human A549 lung epithelial cells. Supplementary Figure S2 shows that the mRNA expression of *TSLP* and *CCL5* in A549 cells induced by co-stimulation with TWEAK and TGF-β1 was not entirely suppressed by DEX treatment (Supplementary Figure S2).

### 2.2. Molecular Mechanisms Resulting in Steroid Unresponsiveness, Induced by the Co-Stimulation of TWEAK and TGF-β1

**Figure 3:**
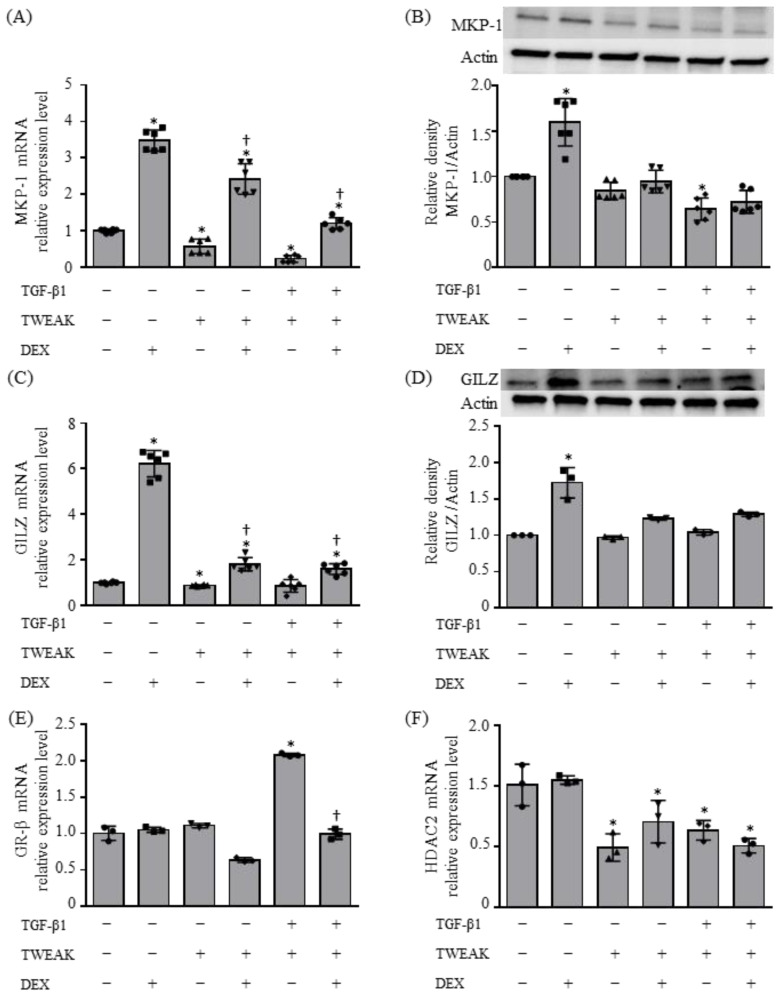
Molecular mechanisms resulting in steroid unresponsiveness, induced by co-stimulation of TWEAK and TGF-β1. The mRNA levels of *MKP-1* (**A**), *GILZ* (**C**), *GRβ* (**E**), and *HDAC2* (**F**) mRNA after 48 h treatment, analyzed by qRT–PCR. MAP kinase phosphatase 1 (MKP-1) and glucocorticoid-induced leucine zipper (GILZ) levels analyzed by immunoblotting (**B**,**D**, upper panel). Densitometric analysis of protein bands (**B**,**D**, lower). Data represent mean ± SD of two independent experiments. * *p* < 0.05, compared to untreated cultures as controls; † *p* < 0.05 compared with cultures in the absence of DEX.

We investigated the expression of MKP-1, GILZ, glucocorticoid receptor beta (GRβ), and histone deacetylase 2 (HDAC2) by the co-stimulation of BEAS-2B cells with TWEAK and TGF-β1. The mRNA and protein expression of MKP-1 and GILZ were upregulated by the addition of DEX, and the DEX-induced upregulation was suppressed by TWEAK alone or co-stimulation of TWEAK and TGF-β1 (Figure 3A–D). The mRNA expression of *GRβ* was upregulated by co-stimulation of TWEAK and TGF-β1 and was suppressed by DEX, and *HDAC2* mRNA was downregulated by TWEAK (Figure 3E,F). Collectively, these findings suggest the involvement of MKP-1, GILZ, and HDA*C2* in the result of steroid unresponsiveness induced by the co-stimulation of TWEAK and TGF-β1.

### 2.3. The Co-Stimulation of TWEAK- and TGF-β1-Induced Steroid Unresponsiveness and the Production of Cytokines Require the Mitogen-Activated Protein Kinase (MAPK) and Nuclear Factor Kappa Beta (NF-κB) Signaling Pathways

**Figure 4:**
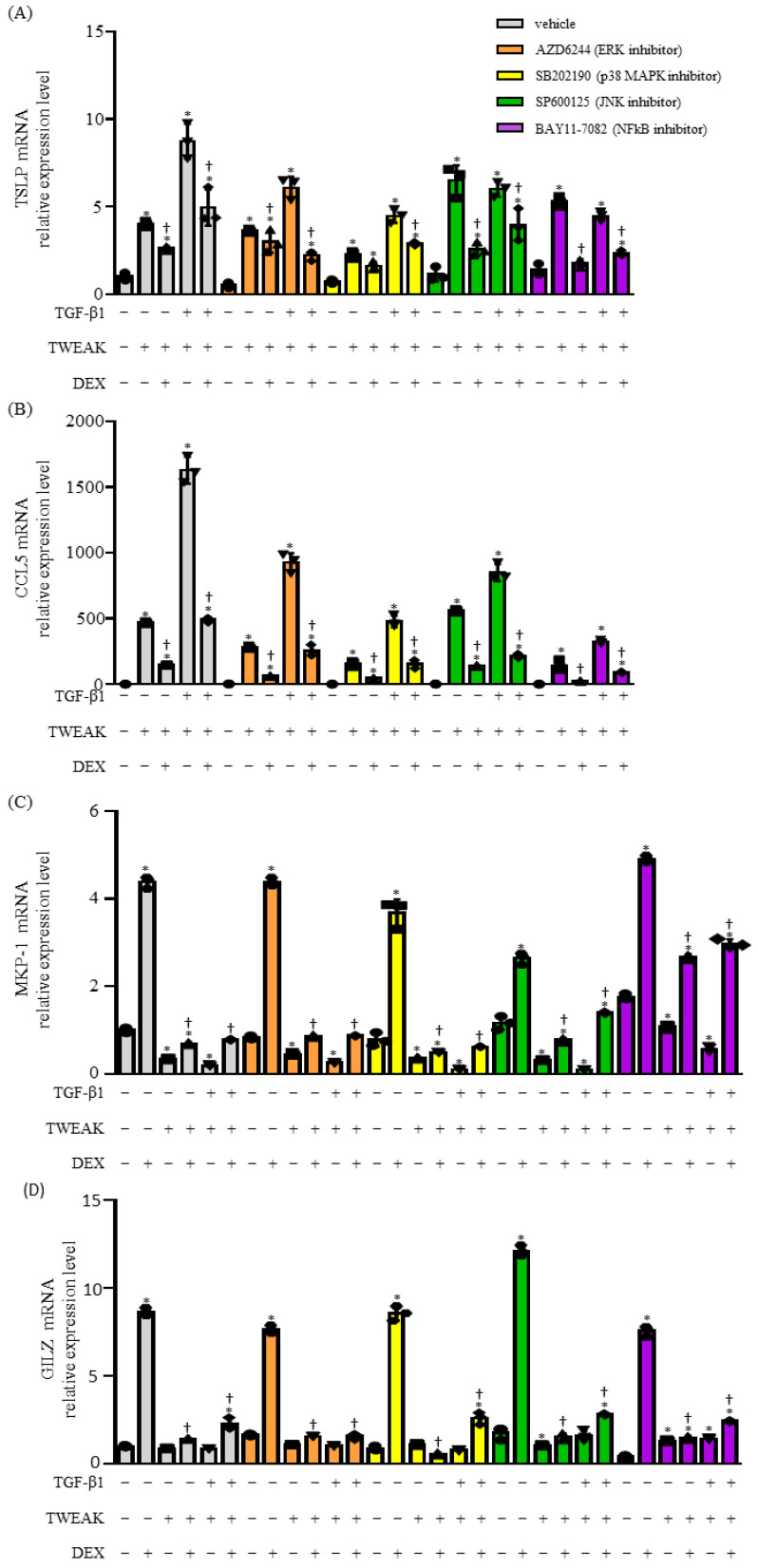
Co-stimulation of TWEAK and TGF-β1 induces steroid-unresponsive production of cytokines through the mitogen-activated protein kinase and nuclear factor kappa beta signaling pathways. Confluent monolayers of BEAS-2B cells were cultured for 48 h in the absence (DMSO as vehicle) or presence of AZD6244 (5 μM), SB202190 (5 μM), SP600125 (5 μM), or BAY11-7082 (2.5 μM) and treated with TGF-β1 (10 ng/mL), TWEAK (100 ng/mL), or TGF-β1 in combination with TWEAK. The mRNA levels of *TSLP* (**A**), *CCL5* (**B**), *MKP-1* (**C**), and *GILZ* (**D**) were analyzed by qRT–PCR. Data represent mean ± SD of two independent experiments. * *p* < 0.05, compared with the vehicle; † *p* < 0.05 compared with cultures in the absence of DEX.

The upregulation of *TSLP* and *CCL5* mRNA, which was not completely inhibited by DEX during the co-stimulation of TWEAK and TGF-β1, was downregulated by treating BEAS-2B cells with specific inhibitors: AZD6244 (an ERK kinase inhibitor), SB202190 (a p38 MAPK inhibitor), SP600125 (a c-Jun N-terminal kinase inhibitor), and BAY11-7082 (an NF-κB inhibitor) (Figure 4A,B). Moreover, SB202190 and SP600125 downregulated DEX-induced *MKP-1* mRNA expression, whereas BAY11-7082 upregulated it (Figure 4C). Remarkably, BAY11-7082 significantly reversed the inhibition of *MKP-1* mRNA expression induced by TWEAK (Figure 4C). *GILZ* mRNA expression was downregulated by AZD6244 and upregulated by SP600125 in the presence of TWEAK, TGF, and DEX (Figure 4D). When expressed as a fold change between the absence and presence of DEX, the addition of an inhibitor, including SB202190, SP600125, and BAY11-7082, to TGF-β1 and TWEAK co-stimulation increased *MKP-1* mRNA expression, but no such change was observed for *GILZ* mRNA (Supplementary Figure S1). These findings suggest that steroid unresponsiveness resulting from co-stimulation of TWEAK and TGF-β1 involves the NF-κB and MAPK pathways.

### 2.4. MKP-1 Is Involved in TSLP Production Induced by Co-Stimulation with TWEAK and TGF-β1 in BEAS-2B Cells

**Figure 5:**
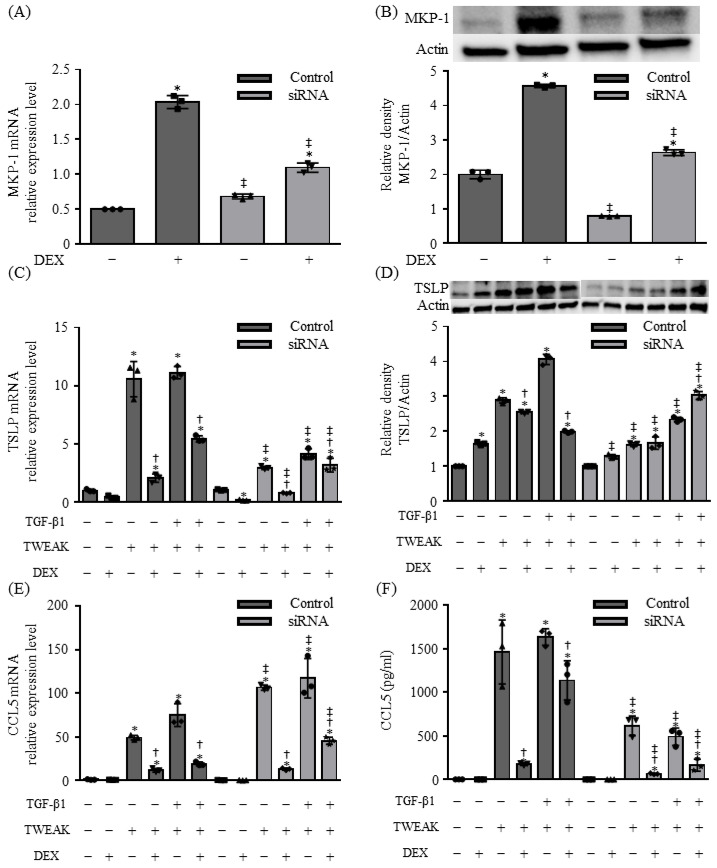
MKP-1 is involved in steroid unresponsiveness induced by co-stimulation with TWEAK and TGF-β1 in BEAS-2B cells. The mRNA levels of *MKP-1* (**A**), *TSLP* (**C**), and *CCL5* (**E**), analyzed by qRT–PCR. Data represent mean ± SD of two independent experiments. MKP-1 and thymic stromal lymphopoietin (TSLP) expression after 48 h of treatment, assessed by immunoblotting (**B**,**D**, upper panel). Densitometric analysis of protein bands (**B**,**D**, lower). The level of CCL5 in cell culture supernatants after 48 h of treatment, analyzed using ELISA (**F**). Data represent mean ± SD of two independent experiments. * *p* < 0.05, compared to untreated cultures as controls; † *p* < 0.05 compared with cultures in the absence of DEX; ‡ *p* < 0.05 compared with control siRNA.

*MKP-1* knockdown using siRNA downregulated DEX-induced *MKP-1* mRNA and protein expression in BEAS-2B cells (Figure 5A,B). MKP-1 knockdown reduced *TSLP* mRNA expression and increased *CCL5* mRNA expression in response to TWEAK and/or TGFβ1 stimulation (Figure 5C,E). However, at the protein level, MKP-1 knockdown resulted in decreased TSLP and CCL5 production (Figure 5D,F). Furthermore, upon the introduction of DEX, the inhibitory effect of DEX on *CCL5* mRNA expression was attenuated, whereas the opposite was observed for *TSLP* mRNA expression (Figure 5C,E). Notably, MKP-1 knockdown abolished the suppressive effect of DEX on TSLP protein production, leading to an increase in TSLP levels, but it did not affect the suppressive effect of DEX on CCL5 protein levels (Figure 5D,F).

## 3. Discussion

Asthma typically manifests as chronic allergic airway inflammation, which involves epithelial damage, repair processes, and remodeling [11,12]. Repeated airway inflammation damages the epithelium, thereby influencing its reparative mechanisms, which ultimately leads to remodeling characterized by thickening of the basement membrane, metaplasia of mucous cells, proliferation of smooth muscle cells and lung fibroblasts, and other associated changes. Although the precise mechanisms of chronic airway allergic inflammatory conditions have not yet been fully elucidated, EMT is considered to play a crucial role in the sequence of events from bronchial epithelial repair to remodeling in individuals with asthma [13,14]. EMT is a fundamental process in embryonic development, tissue remodeling, and wound repair [15]. During EMT, epithelial cells undergo transformation, acquiring enhanced motility by downregulating epithelial markers, such as E-cadherin, and elevating the expression of mesenchymal markers like N-cadherin [16,17]. TGF-β1, a multifunctional cytokine within the TGF superfamily, is upregulated in airway remodeling tissues [13,14,18]. TWEAK promotes NF-κB activation, and the interaction between TWEAK and its receptor Fn14 contributes to proinflammatory effects and EMT in the bronchial epithelium [8,9,19,20]. TWEAK is produced by various cell types, including monocytes, macrophages, dendritic cells, T cells, natural killer cells, and airway smooth muscle cells, and its level is significantly elevated in patients with inflammatory diseases and cancer [19,21]. We have previously demonstrated that TWEAK weakens adhesion factors and enhances TGF-β1-induced EMT in bronchial epithelial cells [8]. Furthermore, both TWEAK and co-stimulation with TWEAK and TGF-β1 induce the production of TSLP and CCL5 during EMT in BEAS-2B bronchial epithelial cells [9]. TSLP mediates type 2 inflammation, including the promotion of activities of type 2 helper T and group 2 innate lymphoid cells [22,23]. CCL5 is a ligand for CC chemokine receptor (CCR) 1 and CCR3 expressed on the surface of eosinophils, contributing to their chemoattractant effect and activity [24,25]. Additionally, the TWEAK level is elevated in airway smooth muscle cells of patients with asthma and in the sputum of children with asthma compared to that of non-asthmatic individuals [21,26]. Notably, in children with asthma, TWEAK expression in the sputum is significantly correlated with the severity and control of asthma, and airway limitation, particularly in cases of non-eosinophilic inflammation [26]. Taking into account these findings and the outcomes of our present study, steroid unresponsiveness in EMT and in the production of TSLP and CCL5 induced by co-stimulation with TWEAK and TGF-β1 is suggested to be a significant mechanism for patients with asthma, which is insufficiently controlled even after treatment with high-dose inhaled or oral glucocorticoids.

Glucocorticoids primarily act by deactivating numerous proinflammatory genes encoding cytokines, chemokines, and adhesion molecules, and various molecular mechanisms contribute to the reduced anti-inflammatory effects of glucocorticoids [1,2,3,4,27,28]. Increased HDAC2 activity and enhanced anti-inflammatory transcription factors and proteins (such as MKP-1 and GILZ) effectively suppress inflammatory genes and proteins. Conversely, an increase in GRβ, which acts as a decoy receptor for glucocorticoid receptors, decreases HDAC2 activity, and the attenuation of MKP-1 and GILZ act in the opposite direction to the effects of steroids [2,27,28,29]. Steroid unresponsiveness is intricately related to epigenetic factors such as the expression of various molecules and HDAC2 activity. Within the group of anti-inflammatory molecules induced by glucocorticoids, notable candidates include MKP-1, GILZ, and an inhibitor of NF-κB (IκBα). Although these molecules are effective in suppressing NF-κB activity, DEX, the steroid in this study, was unable to entirely halt the reduction in TWEAK-induced E-cadherin expression and cytokine production. These findings, in conjunction with the results obtained using various MAPK inhibitors, suggest that TWEAK alone or TGF-β1-co-stimulation-induced steroid unresponsiveness may be mediated through a combination of NF-κB and NF-κB-independent MAPK pathways. Inhibition of the NF-κB pathway notably increased MKP-1 expression, although losing the targets of IκBα, MKP-1, and GILZ. MKP-1 inactivates MAPK and mitigates inflammation by dephosphorylating the threonine and tyrosine residues necessary for MAPK activation [1,2,3,4]. In this study, MKP-1 knockdown decreased *TSLP* mRNA expression and increased *CCL5* mRNA expression in response to TWEAK and/or TGFβ1 stimulation, increasing TSLP production and decreasing CCL5 production at the protein level. Additionally, the inhibitory effect of DEX on *CCL5* mRNA expression was attenuated, while *TSLP* mRNA expression was enhanced. However, MKP-1 knockdown abolished the inhibitory effect of DEX on TSLP protein production, leading to increased TSLP protein levels, but did not affect the inhibitory effect of DEX on CCL5 protein levels. The reason for the opposing effects of MKP-1 knockdown on TSLP and CCL5, as well as the divergence between mRNA expression and protein levels, remains unclear. These conflicting results represent the most significant limitation of this study. Nonetheless, the enhanced TSLP production induced by TWEAK and TGFβ1 stimulation following MKP-1 knockdown was believed to result from the suppression of DEX’s inhibitory effect. This is consistent with a previously reported finding that DEX treatment increased TSLP production in MKP-1-deficient mice with allergic skin inflammation [30]. These results regarding the inhibition of the MAPK pathway are inconsistent, and a comprehensive exploration of the intricate MAPK pathway is essential. However, steroid unresponsiveness induced by either TWEAK alone or co-stimulation with TGF-β1 may involve the NF-κB pathway and MKP-1. Furthermore, the results of MKP-1 knockdown suggested that MKP-1 plays a role in TSLP production during steroid unresponsiveness. However, this is only a partial contribution, and MKP-1 can be considered to be one of the various factors involved. Further experiments using MKP-1-deficient mice are required to explain the exact role of MKP-1 in cytokine production, particularly in TWEAK-related mechanisms of airway inflammation.

Our study has certain limitations. First, we used BEAS-2B cell monolayers, which show the characteristics of undifferentiated basal human bronchial epithelial cells and could not present results for other cell types, such as normal bronchial epithelial cell lines. Second, we could not determine the exact concentration of TWEAK to be used in this study. Although the exact physiological concentration of TWEAK is not yet standardized for experiments on lung diseases, including asthma, stimulation with 100 ng/mL TWEAK was performed in the present study, as previously reported [20,31,32,33,34]. Third, the unresponsiveness of E-cadherin downregulation and N-cadherin upregulation to steroids indicates that EMT induced by this co-stimulation is steroid-unresponsive. However, this study focused on the expression of E-cadherin and N-cadherin. Therefore, verifying other epithelial and mesenchymal markers is imperative for gaining a comprehensive understanding. Finally, no in vivo studies have yet demonstrated the significance of TWEAK signaling in steroid-resistant inflammation in asthma, including this study, highlighting the need for future research in this area.

In conclusion, our study has unveiled that co-stimulation with TWEAK and TGF-β1 results in steroid unresponsiveness in BEAS-2B bronchial epithelial cells, and that this steroid unresponsiveness involve the NF-κB and MAPK pathways, and MKP-1 expression. To the best of our knowledge, this is the first study demonstrating that the downregulation of E-cadherin, upregulation of N-cadherin, and production of TSLP and CCL5 by co-stimulation with TWEAK and TGF-β1 induce steroid unresponsiveness in cultured BEAS-2B bronchial epithelial cells, thereby suggesting that co-stimulation with TWEAK and TGF-β1 may potentially result in steroid-unresponsive airway inflammation.

## 4. Materials and Methods

### 4.1. Reagents

Recombinant soluble human TWEAK and TGF-β1 were purchased from PeproTech (Rocky Hill, NJ, USA). Anti-E-cadherin monoclonal antibody (mAb) (HECD-1) was sourced from Takara (Tokyo, Japan), and anti-N-cadherin mAb (clone32) was procured from BD Biosciences (San Jose, CA, USA). Anti-TSLP mAb (MAB1398) was obtained from R&D Systems (Minneapolis, MN, USA). Anti-MAPK phosphatase-1 (MKP-1) monoclonal antibody (sc-271684) and anti-glucocorticoid-induced leucine zipper (GILZ) mAb (G-5) were purchased from Santa Cruz Biotechnology (Santa Cruz, CA, USA). Anti-β-actin antibody, SB202190, SP600125, and BAY11-7082 were purchased from Wako Chemicals (Osaka, Japan). AZD6244 was obtained from Selleckchem (Houston, TX, USA). Bronchial epithelial growth medium (BEGM) was purchased from Cambrex (East Rutherford, NJ, USA).

### 4.2. Cell Culture

The SV40-transformed normal human bronchial epithelial cell line BEAS-2B and the A549 lung adenocarcinoma cell line were procured from the American Type Culture Collection (Rockville, MD, USA). BEAS-2B cells were maintained in complete BEGM (Lonza, Walkersville, MD, USA), which consists of bronchial epithelial basal medium (BEBM) supplemented with insulin (5 µg/mL), hydrocortisone (0.5 µg/mL), transferrin (10 µg/mL), triiodothyronine (6.5 ng/mL), epinephrine (0.5 µg/mL), human epidermal growth factor (0.5 ng/mL), retinoic acid (0.1 ng/mL), gentamycin (50 µg/mL), and bovine pituitary extract (52 µg/mL). A549 cells were grown in RPMI 1640 (FUJIFILM Wako Pure Chemical Corporation, Osaka, Japan) supplemented with l-glutamine (0.3mg/mL), 10% fetal calf serum, penicillin (100 U/mL), and streptomycin (100 µg/mL). For experiments detailed in Section 2, the culture medium was replaced with fresh BEBM without growth factors and serum, with or without recombinant soluble human TGF-β1 (10 ng/mL), TWEAK (100 ng/mL), or dexamethasone (DEX).

### 4.3. RNA Isolation and Quantitative Real-Time Reverse Transcription–Polymerase Chain Reaction (qRT–PCR)

Total RNA was isolation from BEAS-2B bronchial epithelial cells using an RNeasy Plus Mini Kit (Qiagen, Valencia, CA, USA). RNA was treated with DNase and subjected to cDNA synthesis using a First-Strand cDNA Synthesis Kit (GE Healthcare, Little Chalfont, Buckinghamshire, UK). The qRT–PCR was conducted using a Fast SYBR Green Master Mix (Applied Biosystems, Foster City, CA, USA) on an ABI 7500 Fast real-time PCR instrument (Applied Biosystems, Warrington, UK). Gene-specific primer pairs, detailed in Table 1, were used for qRT–PCR. The comparative threshold cycle (CT) value for glyceraldehyde-3-phosphate dehydrogenase (GAPDH) was used to normalize any variation in loading during qRT–PCR.

### 4.4. Preparation of Cell Lysates and Western Blotting

Whole-cell lysates were obtained using a radioimmunoprecipitation assay buffer. Equal amounts of proteins (10–20 mg) were subjected to sodium dodecyl sulfate–polyacrylamide gel electrophoresis. After electrophoresis, the separated proteins were transferred onto polyvinylidene difluoride membranes blocked with 5% bovine serum albumin in Tris-buffered saline containing 0.1% Tween-20 to prevent non-specific binding. Membranes were incubated overnight with primary antibodies. Specifically, anti-TSLP, anti-N-cadherin, and anti-GILZ mAbs were applied at a 1/500 dilution, while anti-E-cadherin, anti-MKP-1, and anti-β-actin mAbs were used at a 1/1000 dilution. Membranes were further incubated with horseradish-peroxidase-conjugated secondary antibodies at a 1/4000 dilution (GE Healthcare). Signals of protein bands spanning a linearity of four orders of magnitude were captured using a ChemiDoc Imaging System (Bio-Rad Laboratories, Richmond, CA, USA). Subsequently, the acquired data were analyzed using ImageJ image Version 1.54 (National Institutes of Health, Bethesda, MD, USA).

### 4.5. RNA Interference Assay

Lipofectamine RNAiMAX transfection agent (Life Technologies, Inc., Grand Island, NY, USA) along with MKP-1 small interfering RNA (siRNA) and control siRNA (Santa Cruz Biotechnology) were used in this assay. Briefly, the siRNA duplexes and Lipofectamine RNAiMAX were incubated in Opti-MEM I reduced serum medium (Opti-MEM) (Life Technologies, Inc.) at room temperature for 5 min. The siRNA/Lipofectamine RNAiMAX complexes were then transfected into BEAS-2B cells that were subsequently seeded in 6-well plates. After 24 h of incubation at 37 °C, the transfected cells were treated in the absence (control) or presence of TGF-β1 (10 ng/mL), TWEAK (100 ng/mL), or DEX for 48 h. Knockdown efficacy was assessed using qRT–PCR.

### 4.6. Measurement of Cytokine and Chemokine Levels

Culture supernatants collected at 48 h after stimulation were used for measuring the concentrations of CCL5, CCL17, CCL2, and IL-8 using human CCL5, CCL17, CCL2, and IL-8 enzyme-linked immunosorbent assay (ELISA) kits (R&D Systems), according to the manufacturer’s instructions.

### 4.7. Statistical Analysis

Sample normality was assessed using the D’Agostino–Pearson test. For comparisons between multiple groups, one-way repeated-measures analysis of variance (ANOVA) with Tukey’s multiple comparison test, and Friedman’s test with Dunn’s test were used. Statistical significance was set at *p* < 0.05. Statistical analyses were performed using GraphPad Prism v.6 (GraphPad Software, San Diego, CA, USA).

## Figures and Tables

**Table 1:** Gene-specific primer pairs.

Target	Sense Primer (5′ 3′)	Antisense Primer (5′ 3′)
*GILZ*	CTTGGGAGAAGGCCGGAAG	CTGCTCTTGTCAGGGGTCTG
*GRβ*	GGCTATTCAAGCCCCAGCA	TTTGGGAGGTGGTCCTGTTG
*HDAC2*	TCTGCTACTACTACGACGGTGATA	GTCATGCGGATTCTATGAGGCT
*IL-8*	ACTGAGAGTGATTGAGAGTGGAC	AACCCTCTGCACCCAGTTTTC
*CCL2*	CATGAAAGTCTCTGCCGCCC	GGGCATTGATTGCATCTGGCTG
*MKP-1*	CCCTGAGTACTAGCGTCCCT	GGGCCACCCTGATCGTAGAG
*CCL5*	AACCCAGCAGTCGTCCACAG	TTGTTCAGCCGGGAGTCATAC
*CCL17*	CTTCAAGGGAGCCATTCCCC	TACAAAAACGATGGCATCCCTG
*TSLP*	CGCGTCGCTCGCCAAAGAAAT	TGAAGCGACGCCACAATCCTTG
*GAPDH*	GGTCTCCTCTGACTTCAACA	GTGAGGGTCTCTCTCTTCCT

## Data Availability

Data is contained within the article.

## Supplementary Materials

The following supporting information can be downloaded at: https://www.mdpi.com/article/10.3390/ijms252111625/s1.
