# Supplementary material for: Co-Stimulation with TWEAK and TGF-β1 Induces Steroid-Insensitive TSLP and CCL5 Production in BEAS-2B Human Bronchial Epithelial Cells
Source: Int J Mol Sci. 2024 Oct 29;25(21):11625. doi: 10.3390/ijms252111625 (PMC11546882; doi:10.3390/ijms252111625)
Supplement: Supplementary file 1 [file ijms-25-11625-s001.zip › ijms-3142150 R2 Supplementary Figure S1.pdf]

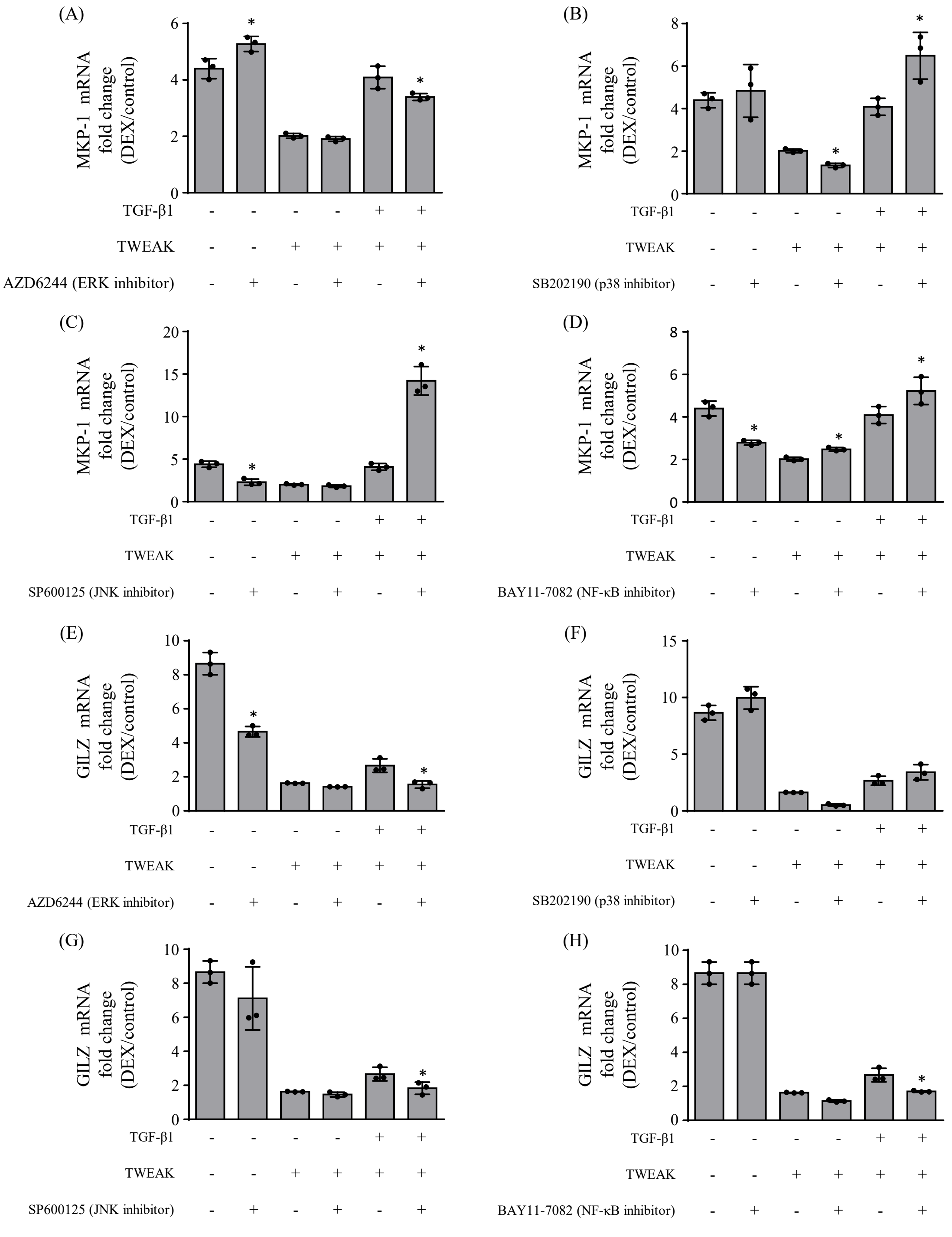

Supplementary Figure S1. Co-stimulation of TWEAK and TGF-β1 induces steroid- unresponsive upregulation of MKP-1 and GILZ mRNA expression through the mitogen-activated protein kinase and nuclear factor kappa beta signaling pathways. Confluent monolayers of BEAS-2B cells were cultured for 48 h in the absence (DMSO as vehicle) or presence of AZD6244 (5 μM) (A and E), SB202190 (5 μM) (B and F), SP600125 (5 μM) (C and G), or BAY11-7082 (2.5 μM) (D and H) and treated with TGF-β1 (10 ng/ml), TWEAK (100 ng/ml), or TGF-β1 in combination with TWEAK. The mRNA levels of MKP-1 (A-D) and GILZ (E-H) analyzed by qRT–PCR. Data represent mean ± SD of two independent experiments. *$p < 0.05$, compared with the vehicle without inhibitor.
