# Supplementary material for: Co-Stimulation with TWEAK and TGF-β1 Induces Steroid-Insensitive TSLP and CCL5 Production in BEAS-2B Human Bronchial Epithelial Cells
Source: Int J Mol Sci. 2024 Oct 29;25(21):11625. doi: 10.3390/ijms252111625 (PMC11546882; doi:10.3390/ijms252111625)
Supplement: Supplementary file 1 [file ijms-25-11625-s001.zip › ijms-3142150 R2 Supplementary Figure S2.pdf]

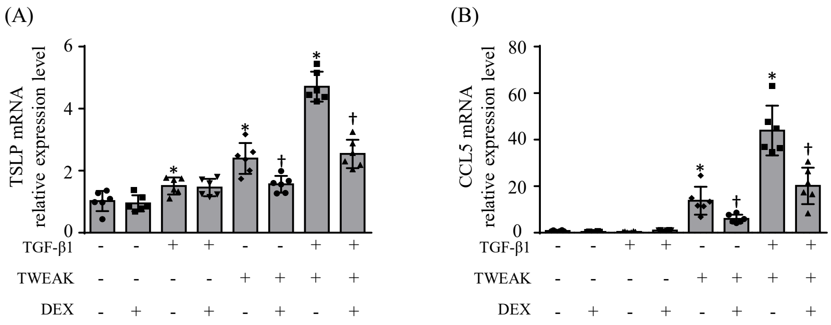

Supplementary Figure S2. Cytokine production induced by co-stimulation with TWEAK and TGF-β1 exhibit steroid unresponsive. The mRNA levels of TSLP (A) and CCL5 (B) after 48 h of treatment analyzed by qRT–PCR. Data represent mean ± SD of two independent experiments. *p < 0.05, compared to untreated cultures as controls; †p < 0.05 compared with cultures in the absence of DEX.
